# The Role of Natural Chalcones and Their Derivatives in Targeting Prostate Cancer: Recent Updates

**DOI:** 10.3390/ijms262412082

**Published:** 2025-12-16

**Authors:** Ola J. Hussein, Dana Elkhalifa, Arij Fouzat Hassan, Feras Alali, Ala-Eddin Al Moustafa, Ashraf Khalil

**Affiliations:** 1 Department of Pharmaceutical Sciences, College of Pharmacy, QU Health, Qatar University, Doha P.O. Box 2713, Qatar; oh1305682@qu.edu.qa (O.J.H.);; 2 Department of Pharmacy, Aspetar Orthopedic and Sports Medicine Hospital, Doha P.O. Box 29222, Qatar; 3 College of Health and Life Sciences, Hamad Bin Khalifa University, Doha P.O. Box 34110, Qatar; 4 College of Medicine, QU Health, Qatar University, Doha P.O. Box 2713, Qatar; 5 Oncology Department, Faculty of Medicine, McGill University, Montreal, QC H3G 2M1, Canada; 6 ABS Research Review & Consultation, Montreal, QC H4L 4P8, Canada; 7 College of Pharmacy, Dubai Medical University, Dubai 19099, United Arab Emirates

**Keywords:** chalcone, prostate cancer, antitumor activities, metastasis, therapy

## Abstract

Prostate cancer (PCa) is the second most prevalent cancer among men and a major cause of cancer-related mortality worldwide. Despite an initial favorable response to hormone-based therapies, many patients ultimately develop an advanced and lethal form of the disease, referred to as castration-resistant PCa (CRPC). CRPC is associated with poor prognosis and a lack of effective curative treatments. As a result, new alternatives or improved therapeutic strategies to combat this life-threatening condition are urgently needed. Chalcones, also referred to as 1,3-diphenyl-2-propen-1-ones, have attracted significant attention because of their potent antitumor properties. Owing to their distinctive chemical structure and diverse biological activities, these compounds are promising candidates for treating various cancers, including PCa. Both naturally occurring and synthetically derived chalcones have demonstrated anticancer potential by modulating key cellular processes, including apoptosis, cell cycle regulation, cell migration, invasion, metastasis and angiogenesis, as well as major signaling pathways, such as PI3K/Akt/mTOR, androgen signaling, and NF-κB. This review aims to outline the recent advances in the therapeutic potential of chalcone derivatives in prostate cancer, with a focus on their molecular targets, mechanisms of action, and translational relevance.

## 1. Introduction

Globally, prostate cancer (PCa) ranks as the second most commonly diagnosed cancer and a major cause of cancer-related mortality in males [1,2]. In 2022, 1,467,854 individuals were diagnosed with PCa, and 397,430 died from the disease [1]. PCa is more common in Western countries, with incidence rates in developed nations being over 25-fold higher than those in developing countries [3].

The onset and development of PCa are associated with several genetic and environmental risk factors, including age, race, genetic mutations, and family history [4]. The risk of PCa increases dramatically with age, where approximately 60% of PCa cases are diagnosed in men older than 65 years [5]. Furthermore, Asians exhibit a lower probability of developing PCa, compared to African Americans, who exhibit a significantly higher incidence and mortality rates, approximately twice that of other ethnic groups [5,6]. Family history is a notable risk factor for PCa, as having a relative with the disease doubles an individual’s likelihood of developing it. The risk increases further if multiple family members are affected or if the diagnosis occurs before the age of 65 [7,8]. Genetic mutations associated with an increased PCa susceptibility are classified as prevalent high-risk mutations and infrequent low-risk mutations [9]. One significant example of a rare but influential mutation occurs in the breast cancer susceptibility gene 2 (*BRCA2*), which is present in approximately 1% of early-onset PCa patients [10]. Carriers of this mutation are 5 to 7 times more likely to develop PCa than non-carriers [9,11].

In recent years, progress in early diagnosis, along with the introduction of new treatments, has significantly improved the overall survival rates for PCa patients. However, despite these improvements, PCa continues to pose a major health burden and remains among the primary causes of cancer-related deaths [12]. For those with metastatic PCa, prognosis remains poor, with a markedly low 5-year survival rate of approximately 30% [5]. Furthermore, more than 20% of individuals diagnosed with localized PCa eventually experience disease recurrence and progression to metastatic PCa, a stage for which curative treatment options remain unavailable [13].

Considering the high prevalence of PCa and the low survival rates in metastatic cases, there is an urgent need for innovative and effective curative therapies. Chalcone derivatives have emerged as a promising therapeutic target for treating PCa and other cancers. Chalcones, also referred to as benzylideneacetophenones, are open-chain flavonoids found in many plant species [14]. Given their attractive biological activities, plants rich in chalcones have been used in folk medicine for centuries [15]. These derivatives exhibit a wide array of biological activities, including anti-diabetic, anti-hypertensive, antiviral and antibacterial effects [16,17,18,19]. Additionally, epidemiological studies on diets rich in polyphenols, such as chalcones, suggest an association with reduced cancer incidence [20]. Consequently, chalcones may hold promise as novel anticancer therapies.

To the best of our knowledge, no comprehensive reviews have focused specifically on the role of chalcone derivatives in PCa. By integrating the most recent research and bringing together various viewpoints, this review aims to address a significant gap in the literature. This study also provides a distinct and valuable perspective on the potential therapeutic opportunities that chalcone derivatives may offer in the management of PCa.

## 2. Prostate Cancer (PCa)

The majority of newly diagnosed untreated PCa patients remain responsive to androgens and depend on androgen for their development [21]. As a result, androgen deprivation therapy (ADT) is the primary therapy for metastatic PCa [22,23]. ADT aims to lower testosterone levels in serum, therefore reducing the binding of androgen to the androgen receptor (AR), which in turn diminishes AR-mediated transcriptional activity. Androgen deprivation can be attained through surgical castration (orchiectomy, which removes the testicles) or reversible medical castration via the use of drugs such as anti-androgens or luteinizing hormone-releasing hormone (LHRH) agonists or antagonists [22,23]. Despite their effectiveness, the response to these therapies is generally temporary, and ultimately, all PCa patients develop resistance, leading to progression to a more advanced and often fatal form known as castration-resistant prostate cancer (CRPC) [24].

Metastatic CRPC (mCRPC) continues to present a significant concern despite the introduction of numerous innovative treatments. The use of chemotherapy for the treatment of mCRPC was established in 2004 with the approval of docetaxel (DTX), a cytotoxic anti-microtubule agent, by the US Food and Drug Administration (FDA) for this purpose [24,25]. While various cytotoxic agents, including 5-fluorouracil, cisplatin, doxorubicin, and capecitabine, have been investigated in earlier trials, none have demonstrated considerable survival benefits, with therapeutic responses observed in only 10–20% of patients [26]. The approval of DTX was based on phase III trials, which revealed a slight survival advantage of up to 3 months for mCRPC patients [27,28]. Since then, DTX has remained the standard first-line therapy for mCRPC and was the sole drug proven to increase survival until 2010. Nevertheless, its survival advantage is temporary, and therapy resistance is unavoidable [25]. Moreover, DTX is a potent drug with a narrow therapeutic index, often resulting in severe side effects such as hypersensitivity reactions, anemia, and neutropenia [29,30].

In the past decade, the FDA has approved a few drugs for the treatment of mCRPC. Despite these advancements, these therapies have not been able to significantly extend overall survival, with improvements generally limited to approximately five months [31,32]. Furthermore, many patients exhibit intrinsic resistance, resulting in a lack of response to these treatments. The newly approved therapies comprise non-endocrine-based agents such as sipuleucel-T, alpharadin and cabazitaxel (CBZ), as well as androgen receptor axis-targeted (ARAT) therapies, such as darolutamide, enzalutamide, abiraterone and apalutamide.

CBZ received approval in 2010 for the management of DTX-resistant mCRPC [33,34]. In 2017, the FIRSTANA trial reported that CBZ was non-inferior to DTX, positioning it as a viable treatment for mCRPC patients with no prior exposure to chemotherapy [35].

That same year, sipuleucel-T became the first immunotherapy to gain approval for the treatment of asymptomatic or minimally symptomatic mCRPC patients [35]. Sipuleucel-T, a cancer vaccine and form of autologous immunotherapy, functions by directing dendritic cells to attack prostatic acid phosphatase, a protein present in 95% of PCa tissues [36,37]. However, its application is restricted to patients with gradually advancing disease, for whom an immediate clinical improvement is not essential [32].

Radium-223 is a recently approved therapy that offers benefits for certain patients with mCRPC. As a calcium-mimetic radioisotope, radium-223 specifically targets metastases in bone tissues, which are the most prevalent site of metastasis in PCa [38]. Consequently, it is recommended only for patients exhibiting symptomatic bone metastases without any indication of visceral metastasis [35]. Although this treatment has a survival benefit of 2 to 4 months, it does not offer a cure, and most patients will eventually develop resistance [31,39,40].

Recent published studies have highlighted the persistent importance of AR signaling in the progression of several mCRPC cases [41]. While ADT can lower systemic androgen levels by 90–95%, it only reduces intra-tumoral androgen levels by approximately 50% [42,43]. In light of these findings and an enhanced comprehension of AR signaling, second-generation ARAT therapies have been developed to attain more effective suppression of AR signaling [44]. Among these, abiraterone and enzalutamide have improved overall survival and have gained FDA approval for the management of mCRPC. Abiraterone works by reducing intra-tumoral androgens through the inhibition of cytochrome P450 17A1 (CYP17A1), an enzyme required for testosterone synthesis [13,45]. Alternatively, enzalutamide is a nonsteroidal anti-androgen that inhibits AR signaling at multiple levels. Like first-generation anti-androgens, enzalutamide functions as a competitive antagonist of the AR by binding to its ligand-binding domain (LBD), preventing androgen attachment. Its binding affinity is 5–8 times stronger than that of earlier anti-androgens [21,46]. Moreover, enzalutamide inhibits AR activity by blocking the translocation of AR to the nucleus and its subsequent attachment to DNA, which in turn suppresses the transcription of tumor-related genes [46]. Both abiraterone and enzalutamide have substantially improved overall survival, extending life by up to 4.8 months [47]. However, up to 40% of patients experience primary resistance and do not benefit from these treatments [48,49]. For patients who respond, the benefits are often short-lived, with secondary resistance developing within a few months. Additionally, the restricted therapeutic alternatives available for refractory PCa patients and the uncertain survival benefits they provide highlight the challenges posed by ARAT agents, which can dramatically alter cancer cell biology, complicating the future management of PCa [50].

The FDA granted approval for rucaparib, a poly (ADP-ribose) polymerase (PARP) inhibitor, in May 2020 to treat mCRPC patients who have BRCA mutations and have previously undergone ADT or chemotherapy [51]. Compared with non-carriers, patients with BRCA mutations represent 5.3% of mCRPC cases and generally experience less favorable outcomes and reduced overall survival [52]. The same month, olaparib, an additional PARP inhibitor, was approved for use in mCRPC patients who have homologous recombination repair (HRR) defects, including individuals with BRCA mutations [53]. Furthermore, in 2017, pembrolizumab, a PD-1 inhibitor, gained approval for use in advanced solid tumors that are associated with mismatch repair deficiencies or microsatellite instability [32,54]. The NCCN guidelines indicate that mismatch repair mutations are present in 2–5% of mCRPC patients [35].

Although therapeutic alternatives for mCRPC have markedly increased over the last 10 years, the disease continues to be largely incurable, with only limited improvements in survival rates. A substantial number of mCRPC patients exhibit resistance to available therapies, and among those who respond, resistance frequently develops in a relatively short time. The prognosis for mCRPC patients remains poor, with a short median overall survival of approximately 1–2 years [32]. Therefore, there is an urgent need for the development of innovative and more effective treatment strategies.

Early strategies focused on targeting alternative pathways in combination with AR inhibitors are being proposed to inhibit the development of aggressive disease forms during PCa treatment [55,56,57]. An increasing number of studies suggest that the current strategy of sequentially using AR-targeted therapies may lead to cross-resistance, extending beyond the specific agents used and potentially affecting all available therapies, including taxanes [57,58,59]. These findings underscore the pressing necessity for innovative medicines targeting other pathways, which could serve as potential treatments for resistant PCa patients or as complementary approaches to improve the efficacy of AR-targeted therapies. Chalcones, a promising class of compounds, are currently under investigation for their potential as multitarget anticancer agents.

## 3. Chalcones

Chalcones, chemically referred to as benzylideneacetophenones or 1,3-phenyl-2-propenones, are a class of open-chain flavonoids widely distributed across numerous plant species. Structurally, chalcones are composed of two phenyl rings joined by a three-carbon α, β-unsaturated carbonyl linker [60]. These compounds can exist in either cis or trans configurations, with the trans isomer exhibiting higher thermodynamic stability, making it the dominant form [61,62].

While chalcones primarily serve as floral pigments in plants, they are also present in the bark, heartwood, fruits, leaves and roots of several plants [60]. They possess a unique chemical structure that enables a broad array of biological activities, including antidiabetic, anti-inflammatory, antimicrobial [17], antioxidant [63], antihypertensive [18], and anticancer properties [64,65]. The diversity of these biological activities is attributed to the distinctive nature of the chalcone structure, which contains substitutable hydrogens, allowing the creation of various derivatives with differing reactivities and specificities toward biological targets [66]. The α, β-unsaturated ketone group in chalcones serves as a Michael acceptor, enabling the formation of covalent bonds with the sulfhydryl groups in proteins, thereby influencing protein functionality [14]. The capacity of chalcones to function as Michael acceptors is determined by the type of electron-withdrawing or electron-donating functional groups present in each derivative, which influences the electron density of the aromatic rings, thereby altering the reactivity of the enone group [14,67]. Chalcones are highly valued by medicinal chemists due to their broad range of biological effects, ease of synthesis, potential for extensive structural modification, minimal engagement with DNA, and low mutagenic risk [68,69]. Consequently, both natural and synthetic chalcones have been extensively investigated in the fields of drug design and discovery.

Natural chalcones act as essential intermediates in the biosynthesis of flavonoids and isoflavonoids, and are naturally produced via the shikimate or acetate metabolic pathways [70]. As secondary metabolites, chalcones play essential roles in plants, contributing to their defense and regulation. These functions include facilitating pollination, protecting against pathogens, shielding against UV radiation, and deterring herbivorous insects [71,72]. Furthermore, chalcones offer notable health benefits and are recognized for their nutraceutical properties, which are linked to a range of significant biological activities [70]. As a result, plants rich in chalcones, such as *Angelica*, *Camellia sinensis* (green tea) and *Piper* have been utilized in folk medicine worldwide for centuries [68,73].

**Figure 1:**
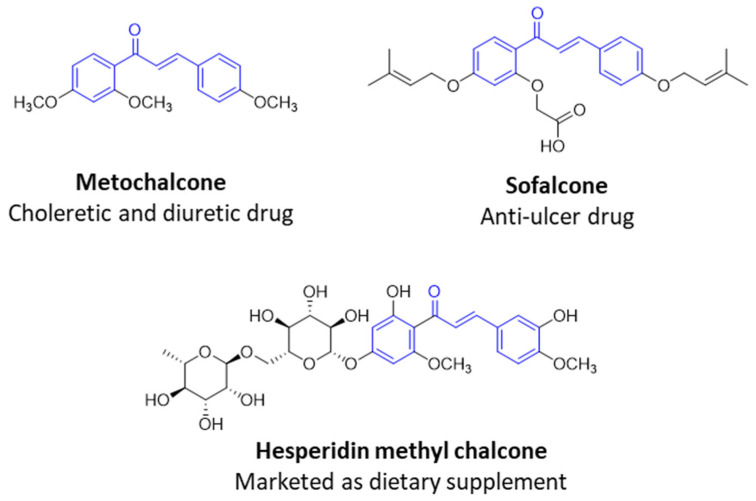
Examples of clinically approved chalcone derivatives. The basic chalcone scaffold is highlighted in blue, while all additional substituent functional groups are shown in black.

Numerous pure chalcones have been extracted from plants and approved for use in humans (Figure 1). One such example is metochalcone, a chalcone derived from *Pterocarpus marsupium*, which has been used as a diuretic and choleretic drug [74]. Similarly, sofalcone, obtained from *Sophora tonkinensis*, is recognized for its anti-ulcer properties, as it promotes mucosal prostaglandin secretion and provides protective effects against *Helicobacter pylori* [75]. Furthermore, hesperidin methyl chalcone has been evaluated in clinical trials for treating venous lymphatic insufficiency and is marketed as part of a combination product with two additional ingredients [76,77].

While plants serve as a primary source of chalcones, the extraction of these compounds from natural sources poses significant challenges, often involving complex procedures that yield low quantities. Consequently, the chemical synthesis of chalcones offers a practical alternative to the resource-intensive natural product extraction. This approach simplifies the process, reduces time and cost, and enhances yield, enabling the production of a variety of chalcone analogs that may not occur naturally.

Chalcones are commonly synthesized via the Claisen-Schmidt condensation reaction, wherein a substituted benzaldehyde reacts with a substituted acetophenone under either basic or acidic conditions [64]. To increase yields and promote environmentally sustainable practices in chalcone synthesis, alternative synthetic methods to the traditional Claisen-Schmidt condensation have been proposed. These include the Suzuki Miyaura reaction, ultrasound-assisted synthesis, microwave-assisted synthesis and the Friedel-Crafts method [78]. Additionally, eco-friendly synthesis techniques such as solvent-free approaches have also been explored [70,79].

The effective utilization of natural chalcones as remedies for numerous conditions has motivated medicinal chemists to design new chalcone analogs with enhanced biological activities. Traditionally, modifications to the phenyl rings have been the main strategy for structural alterations [66]. By replacing one or more protons on the aromatic rings with different functional groups such as aryl, alkyl, halogen, nitro, amino and carboxylic groups, a range of chalcones can be synthesized [66]. The nature and position of these functional groups strongly influence their biological effects on different targets, mirroring the activity of naturally occurring chalcones [80].

Recent progress in chalcone design has resulted in the development of chalcone hybrids, in which the traditional phenyl rings are substituted with heterocyclic rings or other structural frameworks [81]. This strategy is rooted in molecular hybridization, which combines the pharmacophoric moieties of different active compounds to create new hybrids with enhanced pharmacological effects, selectivity, pharmacokinetic features, safety profiles, and minimal susceptibility to therapeutic resistance [82]. A range of hybrid chalcones including pyrrole-, coumarin-, pyrimidine-, tetralone-, and pyridine-, based derivatives have been synthesized by connecting the chalcone scaffold with compounds known to exhibit anticancer properties. These hybrids have shown either additive or synergistic pharmacological effects [81]. Molecular hybridization has demonstrated success as a valuable approach for discovering new derivatives with enhanced anticancer activity, positioning hybrid molecules as a promising avenue in modern drug discovery.

## 4. Chalcones and PCa

**Figure 2:**
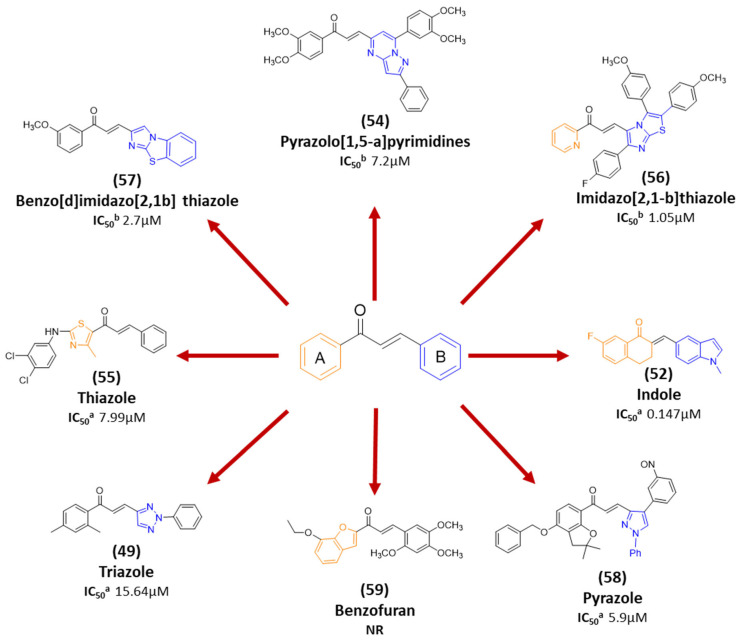
Summary of heteroaromatic based chalcone derivatives tested in prostate cancer (PCa). Numbers in parentheses indicate the compound numbers as listed in Table 1, and the corresponding IC_50_ values shown in the figure are derived from the original studies summarized in Table 1.

Many chalcones, whether synthetically or naturally derived, have shown promising anticancer properties by targeting signaling cascades and processes involved in various stages of PCa development. Chalcones influence multiple biological functions, such as cell proliferation, angiogenesis, apoptosis, the cell cycle, and metastasis. The antiproliferative activity of these compounds is often associated with their impact on signaling pathways associated with carcinogenesis, such as the β-catenin/Wnt, nuclear factor kappa B (NF-κB), and PI3k/Akt/mTOR pathways. In addition, chalcones demonstrate cytotoxicity against metastatic cancer cells, including those resistant to conventional therapies [83]. As chalcones are found in various edible plants, including kava (*Piper methysticum*), licorice (*Glycyrrhiza* spp.), hops (*Humulus lupulus*) and hop-derived products, as well as several fruits, vegetables and spices, their ability to target multiple pathways suggests that they may possess a relatively broad therapeutic window [80,84]. Chalcone analogs could thus emerge as effective anticancer agents, offering favorable efficacy and safety profiles for PCa treatment (Figure 2). Population-based studies further support this idea, revealing a significant negative correlation between cancer occurrence and the use of chalcone-rich kava plant extract [85]. Table 1 presents studies evaluating the effects of chalcone derivatives on different PCa models. The key cellular functions and signaling pathways that chalcones may target in PCa are outlined below.

### 4.1. Apoptosis

Apoptosis, commonly known as programmed cell death, is a precisely controlled biological process critical for sustaining tissue homeostasis by eradicating dysfunctional or aged cells [86]. When this defense mechanism is impaired, it disrupts the equilibrium between cellular survival and apoptosis, providing conditions that promote malignant transformation and tumor progression [87]. Consequently, evasion of apoptosis is recognized as a hallmark of cancer and a major contributor to treatment resistance. The execution of apoptosis primarily depends on the activation of cysteine proteases called caspases, which are activated via two main pathways: the intrinsic (mitochondrial) and the extrinsic (death receptor) pathways [88]. The extrinsic pathway begins with the interaction of death receptors on the cell surface with pro-apoptotic ligands, such as Fas ligand (FasL), the tumor necrosis factor-α (TNFα) and TNF-related apoptosis-inducing ligand (TRAIL) [89]. In contrast, the intrinsic pathway is initiated by the release of cytochrome c from the mitochondria, a process regulated by the Bcl-2 family of proteins [90]. Members of this protein family are divided into pro-apoptotic (e.g., Bax, Bcl-xS, Bad, Bim and Bid) and anti-apoptotic (e.g., Bcl-XL, Bcl-2, and Mcl-1) categories [88]. Despite these distinct signaling cascades, both pathways often involve caspase-3 activation, resulting in the breakdown of critical cellular structures such as cytoskeletal proteins and DNA, ultimately causing cell death [91].

Dysregulation of apoptotic proteins is frequently observed in tumors, enabling the evasion of apoptosis. In PCa, these disruptions are associated with ADT resistance and the progression to more aggressive disease stages [92,93]. For instance, Bcl-2 is often overexpressed in androgen-independent PCa, facilitating cell survival in environments with low androgen levels [90]. Conversely, the expression patterns of caspase-1 and caspase-3 are significantly lower in PCa tissues than in normal prostate tissue [94]. Additionally, PCa cells often resist TRAIL-induced apoptosis because of genetic alterations in the 8p21-22 chromosomal region, which harbors the gene encoding the TRAIL receptor DR5, or because of elevated levels of anti-apoptotic proteins [95,96]. Since many anticancer therapies rely on triggering apoptosis to destroy cancer cells, disruptions to these signaling pathways allow malignant cells to avoid cell death, promoting uncontrolled growth and resistance to treatments [89]. However, these deficiencies in apoptotic pathways may also present opportunities for developing innovative cancer therapies, making them promising areas for targeted intervention [97].

**Figure 3:**
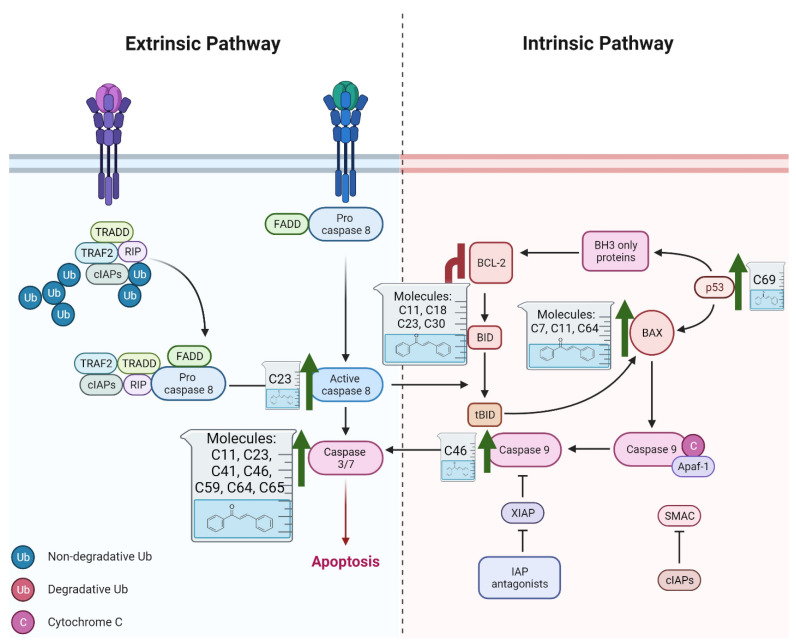
Role of Chalcones in modulating intrinsic and extrinsic apoptosis pathways in prostate cancer (PCa). Compound numbers shown in the figure correspond to the numbering used in Table 1. Arrows indicate activation or signaling progression, whereas T-shaped bars indicate inhibition or suppression of the indicated target.

Chalcone derivatives have demonstrated considerable promise in selectively inducing apoptosis in PCa cells by targeting distinct apoptotic signaling pathways (Figure 3). Many chalcones disrupt the mitochondrial membrane potential, effectively triggering apoptosis [98,99,100]. This disruption is frequently linked to the activation of caspase-3 and caspase-9 [101], upregulation of pro-apoptotic proteins such as Bid and Bax [102,103], and downregulation of anti-apoptotic proteins such as Bcl-xl and Bcl-2 [99,102]. Additionally, some chalcones were shown to trigger apoptosis through the extrinsic pathway, specifically by enhancing TRAIL-induced apoptosis [104,105,106,107]. TRAIL is an endogenous cytokine that exhibits anticancer activity and selectively targets cancer cells for apoptosis while sparing healthy cells [108]. However, TRAIL resistance is observed in certain PCa cell lines [104]. Notably, 2-hydroxy-4-methylsulfonyl chalcone has demonstrated the ability to effectively restore sensitivity to TRAIL-induced apoptosis in resistant PCa cell lines [106]. This sensitization was mediated by enhanced activation of the TRAIL death receptor (DR5) and reduced expression of Bcl-2 [106]. Similarly, another study demonstrated a synergistic activity between the prenylated chalcone, xanthohumol, and TRAIL in triggering apoptosis in LNCaP PCa cell line [102]. Individual treatments with xanthohumol or TRAIL induced apoptosis rates of 11.1% and 12.57%, respectively; however, their combination markedly enhanced apoptosis, up to 76.58% [102]. Collectively, these results underscore the potential use of chalcones as both standalone and adjuvant therapeutic agents for PCa, either by directly inducing apoptosis or by promoting the response of resistant PCa cells to other treatments.

### 4.2. Cell Cycle

The cell cycle is a structured and tightly regulated process essential for cellular growth and division. It comprises four sequential phases: Gap 1 (G1), DNA synthesis (S), Gap 2 (G2), and mitosis (M), with checkpoints built into each phase to control growth and maintain the integrity of genetic material [109]. The progression of the cell cycle is primarily directed by cyclin-dependent kinases (CDKs) and their associated cyclins. CDKs are kinases that pair with cyclins to phosphorylate key proteins, promoting progression through the cell cycle phases [110]. Cyclin-CDK complexes are carefully controlled by cyclin-dependent kinase inhibitors (CDKIs) such as P21CIP1, P27KIP1, and P57KIP2, which function as negative regulators of CDK activity [111]. CDK activity is stimulated by mitogenic signals and inhibited when DNA damage is sensed via cell cycle checkpoints [109]. Checkpoints then activate inhibitory signals to halt the cell cycle, allowing time for the damage to be repaired. If the damage is irreparable, cells are directed toward senescence or programmed cell death [112]. Dysregulation of cell-cycle control mechanisms disrupts these regulatory checkpoints, leading to unrestrained cell division and the development of neoplastic transformations [112]. These aberrations are fundamental to cancer progression, as they allow cells to bypass normal growth controls and proliferate uncontrollably.

Nearly all malignancies involve direct or indirect disruptions to the cell cycle, making these alterations a hallmark of cancer [113]. Abnormalities in cell cycle regulation have been linked to almost all regulatory proteins that are involved in this process [112]. For example, Cyclin D1 is notably overexpressed in androgen-independent PCa, and is associated with tumor metastasis [114,115,116]. Similarly, the absence of p27 (a key CDK inhibitor) is observed in around 16–68% of patients diagnosed with PCa and strongly correlates with enhanced tumor development [117]. Owing to the common occurrence of cell cycle dysregulation in PCa and its critical role in tumors, targeting the cell cycle process represents a promising strategy for novel cancer therapies. These therapeutic approaches could address the uncontrolled proliferation that underpins cancer progression, offering hope for more effective PCa treatments.

**Figure 4:**
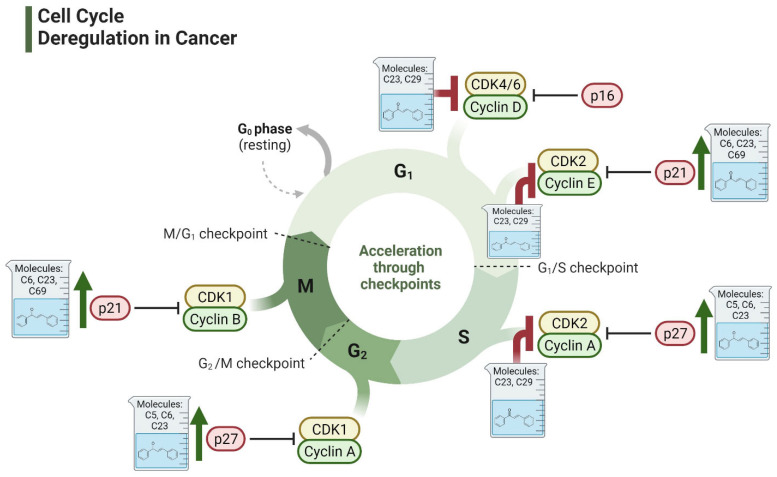
Effect of chalcones on modulating the cell cycle and its regulators in PCa cells. Compound numbers shown in the figure correspond to the numbering used in Table 1. Arrows indicate activation or signaling progression, whereas T-shaped bars indicate inhibition or suppression of the indicated target.

Studies have consistently demonstrated that the antiproliferative activity of chalcones against PCa cells is repeatedly linked to their impact on cell cycle regulation (Figure 4) [83,118,119,120,121,122]. For example, licochalcone-A has been found to decrease cyclin B1 and Cdc2 levels in PC3 cell line, resulting in G2/M phase cell cycle arrest [123]. Similarly, a chalcone derivative containing dithiocarbonates induced G2/M arrest by upregulating the CDK inhibitor p21, while downregulating cyclin B1 and CDK1 [124]. Although G2/M arrest is the predominant effect of most chalcone derivatives, some studies have reported G1 phase arrest. For example, Sun et al. have shown that a methoxy-chalcone analog elicited time- and concentration-dependent G1 phase arrest in PC3 cell lines [125]. This was associated with a decrease in multiple G1 modulators, including cyclin E, cyclin D1, CDK2, CDK4, phosphorylated retinoblastoma (Rb) protein, Cdc25A, and E2F-1 [125]. Interestingly, the effects of chalcone derivatives on the cell cycle can vary depending on the cell line or treatment duration. Isoliquiritigenin was observed to result in G1 phase arrest in DU145 cells after 2 h of treatment, shifting to G2/M arrest after 4 h [126]. Furthermore, a methoxy chalcone derivative caused G2/M arrest in cell lines harboring a p53 mutation but induced G0/G1 arrest in cell lines with wild-type p53 [127]. Together, these results indicate that, irrespective of the specific cell cycle phase targeted, chalcone-induced cell cycle arrest consistently culminates in apoptosis.

One potential mechanism by which chalcones block cell cycle progression is through their binding to tubulin and the subsequent disruption of microtubules [66,128]. Like other antimitotic agents, chalcones can impact microtubules by either inhibiting tubulin polymerization comparable to colchicine, or via stabilizing microtubules and promoting polymerization similar to taxanes [129]. In fact, using chalcones as inhibitors of microtubule assembly is one of the earliest therapeutic applications evaluated for these compounds [129]. Several studies have demonstrated that chalcones inhibit tubulin polymerization through binding to the colchicine binding site, as confirmed through docking analyses [130,131,132]. A library of 1,2,3-triazole-based chalcones was developed and assessed their cytotoxic effects on DU145 PCa cell line [131]. These derivatives significantly suppressed tubulin polymerization and promoted the arrest at the G2/M phase. In contrast, other reports revealed that chalcones can enhance tubulin polymerization. For example, an isoprenylated chalcone derived from *Dalea frutescens* was found to exhibit antitumor activity against AR-positive (AR+) PCa cells [133]. Mechanistic studies revealed that this compound increased tubulin polymerization rates and caused G2/M phase arrest [133]. Regardless of whether chalcones inhibit or promote tubulin polymerization, their interaction with tubulin consistently leads to G2/M phase arrest and antiproliferative activity, underscoring their potential as antimitotic agents.

### 4.3. Cancer Cell Invasion and Migration

Typically, localized PCa is associated with favorable prognosis; however, the 5-year survival rate declines sharply to 30.2% once the disease progresses to metastatic stages [5]. Metastatic cancer spreads through several steps: breaking tight cell junctions, detaching from the original cancer location, migrating to nearby tissues, invading blood or lymphatic vessels, exiting these vessels (extravasation), and metastasizing to distant sites [134]. At the molecular level, epithelial-to-mesenchymal transition (EMT) plays a critical role in facilitating metastasis [135]. During EMT, epithelial cells undergo significant morphological alterations, transitioning from a cuboidal to a spindle-shaped form [136]. This process is characterized by the upregulation of mesenchymal markers, such as N-cadherin, fibronectin, and vimentin, alongside the downregulation of epithelial markers, such as E-cadherin and occludins [137]. These changes result in the loss of cell–cell adhesion and an increase in stem-like properties, which promote the invasive behavior necessary for metastasis [138]. EMT is regulated by several signaling pathways, such as the TGF-β, EGF, Wnt/β-catenin, and NF-κB pathway, either directly or indirectly [136,138].

Several chalcone derivatives have been shown to inhibit EMT and, as a result, reduce the invasion of various cancer types [64,139,140]. While the role of chalcones in modulating EMT in PCa remains relatively underexplored, one study documented their ability to suppress EMT-associated metastatic behavior in PCa [124]. They investigated a new set of dithiocarbonate-based chalcones and reported that they suppress cancer cell migration and EMT by increasing E-cadherin expression, reducing N-cadherin, and suppressing vimentin, MMP2, and MMP9 in PC3 cell lines [124]. Although not directly investigated, EMT inhibition could be an important mechanism in underlying the antiproliferative activity of chalcones against PCa. Various chalcone derivatives have been demonstrated to reduce PCa cell invasion and migration by impacting EMT-related pathways and effectors, including TGF-β, VEGF, MMP, and NF-κB [141,142,143]. These studies collectively indicate that chalcones are capable of preventing PCa cell migration, invasion, and metastasis by suppressing EMT.

### 4.4. PI3k/Akt/mTOR Pathway

The PI3K/AKT/mTOR signaling pathway plays crucial roles in regulating cell metabolism, survival, and proliferation [144]. Elevated activity of this pathway has been reported in PCa, with a particularly significant upregulation observed in CRPC [145,146]. Alterations in PI3K pathway genes have been reported to be highly prevalent in PCa, occurring in approximately 42% of primary and 100% of metastatic PCa cases [147,148,149]. Therefore, chalcones have emerged as promising therapeutic agents for targeting the PI3K/AKT/mTOR signaling pathway in PCa [100,106,125,150,151,152,153]. For example, a chalcone molecule with methyl sulfonyl and hydroxy substituents has been demonstrated to overcome TRAIL resistance in PCa cell lines. The compound enhanced TRAIL-induced apoptosis through the downregulation of Bcl-2, PI3K/AKT, COX-2, and NF-κB signaling pathways [106]. Similarly, isoliquiritigenin, another chalcone, has been reported to modulate PI3K/AKT/mTOR cellular function, particularly by diminishing the expression of the *ErbB3* gene [150]. This inhibition prevented the phosphorylation of ErbB3 and reduced the engagement of the p85 subunit of PI3K, which in turn inhibited Akt phosphorylation [150]. While these findings are promising, they are limited by the lack of adequate in vivo studies. Therefore, further in vivo research is necessary to validate these mechanisms of action and to evaluate their therapeutic potential in PCa management.

### 4.5. Angiogenesis Pathway

Angiogenesis is the formation of new blood vessels from existing blood vessels. Uncontrolled angiogenesis is a key driver of cancer progression and metastasis [154]. Cancer cells can provoke angiogenesis by disrupting the physiological balance of pro-angiogenic and anti-angiogenic signals, thus facilitating the growth of new blood vessels to supply the growing tumors with oxygen and nutrients [155]. Vascular endothelial growth factor (VEGF) is a key activator of angiogenesis, which enhances proliferation of endothelial cells and increases vascular permeability [156]. In PCa, the overexpression of VEGF has been linked to enhanced metastasis and resistance to therapy [157,158]. Owing to its pivotal role in tumor growth and spread, angiogenesis is considered one of the hallmarks of cancer, making angiogenesis inhibition a promising therapeutic target for cancer.

Numerous antiangiogenic therapies have been clinically tested for therapeutic management of PCa; nonetheless, the outcomes have been inconclusive and unsatisfactory [159]. For instance, the addition of bevacizumab to hormone therapy in hormone-sensitive PCa has been linked to improved relapse-free survival, highlighting the role of angiogenesis in PCa progression [160]. Nevertheless, overall survival did not improve when a combined therapy of bevacizumab, prednisone and DTX was used in CRPC, and this combination was associated with an increased rate of treatment-related deaths [161]. Similarly, other antiangiogenic agents, such as aflibercept, sunitinib, and lenalidomide, have not been shown to improve overall survival in patients with CRPC patients [162,163,164]. Additionally, antiangiogenic therapies are frequently used in conjunction with other treatments, as they primarily limit tumor growth by inhibiting blood vessel formation rather than directly eradicating the tumor. Thus, further research should explore novel antiangiogenic therapies that are more effective and safer for combating cancer by targeting multiple mechanisms.

As multi-target agents with exceptional antiangiogenic effects, chalcones have the potential to serve as alternatives to current antiangiogenic therapies. A chalcone derivative, 3,4,2′,4′-tetrahydroxychalcone, was shown in one study to significantly suppress angiogenesis by attenuating the expression of VEGF and MMP-9 [141]. In our laboratory, we explored the antiangiogenic activity of newly developed potent chalcone analogs and found that their antitumor activity was associated with the potent suppression of angiogenesis, as evidenced by the in ovo chicken embryo chorioallantoic membrane (CAM) model [64,165]. Similarly, other investigators designed a new 3′,5′-diprenylated chalcone and reported that it substantially downregulated VEGF, a key player in the modulation of angiogenesis, in PCa cell lines [142]. Furthermore, this chalcone reduced the growth of PCa cells in vivo, suggesting that its antiangiogenic effects contribute to its anticancer activity [166]. Collectively, these findings support the idea that chalcones possess promising antiangiogenic therapeutic potential for PCa.

### 4.6. Androgen Receptor Signaling

Mutations or alterations in AR are central to the development and progression of PCa [167]. Thus, targeting AR through blockade by specific drugs has the potential to inhibit PCa progression. However, resistance to these therapies often develops after several years, leading to the progression of CRPC. In CRPC, cancer cells adapt to survive at low androgen levels through mechanisms such as AR overexpression, AR point mutations, alterations in androgen biosynthesis, and changes in androgen cofactors [168]. Therefore, targeting the AR remains critical for treating PCa, especially in hormone-refractory or advanced stages.

Several studies have explored the potential of chalcones in targeting the AR in PCa [169,170,171]. Jackson et al. investigated the antitumor activities of dibenzoylmethane, a chalcone analog, and found that it significantly downregulated AR protein and gene expression in a dose-dependent manner. Furthermore, this compound suppressed the secretion of prostate-specific antigen (PSA), a tumor marker regulated by AR [169]. A similar effect was observed with another chalcone derivative, isoliquiritigenin, further confirming chalcones as potential AR-targeting agents [170]. Moreover, an ionone-based synthetic chalcone demonstrated potent inhibitory effects (IC_50_ of 0.74 μM) in the LNCaP PCa cell line and successfully blocked dihydrotestosterone (DHT) from activating the wild-type AR. Notably, this compound also exhibited activity against clinically relevant AR mutations, including W741C, H874Y, and T877A [171]. These data collectively indicate that chalcone analogs have potential as treatments for AR-naïve, mutant and advanced forms of PCa.

### 4.7. Inflammatory Pathways

The link between inflammation and PCa development and progression has been extensively studied. Altered levels of reactive oxygen species (ROS), cytokines, chemokines, and other transcription factors have been linked to the initiation and growth of various malignancies, including PCa [172,173]. In particular, inflammation and oxidative stress play crucial roles in regulating the AR, a key receptor involved in the progression of PCa to CRPC [174,175]. Additionally, NF-κB activation is closely linked to inflammation and plays a significant role in PCa tumorigenesis and the development of CRPC [176,177]. Specifically, NF-κB transcription factors were found to enhance cell proliferation, invasion, and survival [176,177]. Among the various NF-κB subunits, p52, RelA, RelB, and c-Rel have been shown to be particularly important in PCa [178]. Additionally, several NF-κB target genes, such as VEGF, caspase-8, Bcl-2, Bax, and MMP-9, have been implicated in the pathogenesis of PCa [177]. Numerous natural and synthetic chalcone-based compounds have been shown to target NF-κB signaling [100,141,179,180]. Among these, butein has been demonstrated to suppress NF-κB activity by downregulating the expression of MMP-9 and VEGF. This compound also induced G2/M phase cell cycle arrest [141]. Since these target genes are also involved in angiogenesis and metastasis, these results indicate that chalcone derivatives may act as effective therapeutic agents for inhibiting angiogenesis, cell growth, and invasion in PCa.

### 4.8. Cancer Stem Cells

Physiologically, embryonic and adult stem cells are characterized as a small population of cells capable of self-renewal, differentiation, and the reconstitution of various tissues into mature cell types that constitute each organ. In cancer, a similar rare subpopulation of cells, known as cancer stem cells (CSCs), has been identified. These CSCs exhibit self-renewal capabilities and stem cell-like properties, which are crucial for cancer initiation, progression, resistance to therapy, recurrence, and metastasis [181,182]. Several signaling pathways that are involved in regulating normal stem cell behavior, were reported to be frequently deregulated in cancers [181]. Among these pathways are NF-κB, Wnt/β-catenin, TGF-β, and Hedgehog [183,184].

Studies on PCa have demonstrated that prostate cancer stem-like cells (PCSCs) are characterized by low or absent AR and PSA expression. These cells contribute to both the initiation of PCa and its progression to CRPC [185]. Given the role of PCSCs in PCa, targeted therapies against these cells represent a promising strategy for the eradication of PCa. One chalcone analog, 2′-hydroxy-2,4,4′,5,6′-penta-methoxychalcone, has been explored for its effects on PCSCs, particularly in combination with taxane therapy for both PCa and taxane-resistant (TXR) PCa [186,187]. This compound significantly enhanced the effects of paclitaxel and DTX when used together in the treatment of PCa and TXR PCa. Wen et al. reported that this molecule sensitized paclitaxel-resistant PCa cells to paclitaxel by upregulating the expression of *miR-34a* (a tumor suppressor gene) and reversing the expression of its downstream target genes [186]. The combination of paclitaxel and chalcone effectively inhibited the proliferation, migration, and growth of PCSCs, highlighting the role of chalcones in targeting CSCs. When this combination was loaded into micelles, PC3-TXR PCa cell viability decreased as the paclitaxel concentration increased. In addition, treatment of PC3 and PC3-TXR cells with paclitaxel resulted in IC_50_ values of 55.6 and 2580 nmol/L, respectively. However, dual therapy improved these effects, reducing the IC_50_ to 49.8 and 93.2 nmol/L, respectively, demonstrating the ability of chalcones to reverse paclitaxel resistance. Compared with paclitaxel monotherapy, in vivo testing of the micelle-based dual therapy significantly inhibited prostate tumor growth in nude mice. This effect was linked to the reversal of SIRT1, cyclin D1, E-cadherin, and miR-34a expression [186]. These results emphasize the potential of chalcone derivatives in reducing chemoresistance to conventional therapies and inhibiting PCSC growth.

## 5. Toxicity and Safety Profile of Chalcones

Despite the extensive evidence supporting the anticancer potential of chalcone derivatives, their toxicity and overall safety profile remain inadequately characterized. Among the studies reporting the anticancer effects of chalcones in PCa models, only a limited number have directly compared their cytotoxicity in cancer cells versus normal cells. The reported selectivity varies markedly depending on the specific derivative and the experimental model used. While some studies describe a high selectivity index, with certain derivatives exhibiting more than 100-fold selectivity toward cancer cells [188], others demonstrated only moderate or no selectivity (Table 1) [189,190]. Similarly, although the in vivo antitumor potential of chalcones has been extensively investigated, data on their systemic toxicity are still limited [191]. In a breast cancer xenograft model, Li et al. reported a favorable safety profile for a quinoline-chalcone derivative with a median lethal dose (LD_50_) of 665.62 mg/kg, while a dose of 20 mg/kg reduced tumors by 68.9% without observable toxic effects [192,193]. On the other hand, Zhou et al. reported severe liver damage upon oral consumption of flavokawain B in BALB/C mice [194], whereas dietary feeding with flavokawain A did not induce detectable adverse effects or organ dysfunction. Therefore, more comprehensive in vivo toxicological studies, including LD_50_ determination and acute toxicity assessment, are needed to better define the therapeutic window of promising chalcone analogs, particularly in PCa models.

Interestingly, several strategies designed to enhance the selectivity of chalcones have been reported and show promise in improving their anticancer potential. Structural modifications, particularly in the α,β-unsaturated carbonyl moiety, can markedly influence the selectivity [195,196,197]. Additionally, nanoparticle-based delivery systems have been employed in a few studies to encapsulate poorly soluble chalcones, enabling lower effective doses and decreased exposure of normal tissues [198,199]. Other targeted delivery strategies, such as glycosyl conjugates, where shown to exploit overexpressed transporters and receptors on cancer cells to enhance selective uptake of chalcone conjugates [200,201]. Collectively, these approaches offer promising avenues to enhance antitumor efficacy and reduce systemic toxicity and should be more systematically explored for PCa–directed chalcones.

## 6. Insights into Structural Perspectives

Among the 73 lead analogs covered in this review (Table 1), 19 are of natural origin, whereas the remainder are synthetic derivatives. Particularly, butein, isoliquiritigenin, flavokawain A, flavokawain B, cardamonin, licochalcone A, xanthohumol, and isobavachalcone are among the most frequently investigated anticancer chalcone derivatives. Natural chalcones with reported anticancer activity typically incorporate hydroxy groups and/or prenyl/isoprenyl substituents and methoxy groups. Both hydroxy and methoxy groups have been shown in multiple studies to modulate anticancer activity [202,203], whereas prenyl substituents were shown to substantially increase lipophilicity, potentially enhancing cellular uptake [204,205]. Additionally, natural chalcones may exert anticancer effects either directly or indirectly through their metabolites. For example, xanthohumol can undergo isomerization to isoxanthohumol in the stomach [206], and isoxanthohumol itself has also been reported to display anticancer activity [207]. On the other hand, synthetic chalcones encompass a broader range of electron-donating and electron-withdrawing groups, as well as diverse heteroaromatic rings, including pyrimidine, thiazole, indole, pyrazole, triazole, and benzofuran moieties. Owing to the variability in cytotoxicity assays, cell lines, and exposure times used across studies, a rigorous quantitative SAR analysis was not feasible in this review. Nevertheless, synthetic chalcone derivatives have repeatedly succeeded in lowering the half-maximal inhibitory concentration (IC_50_) values against PCa cell lines to the low micromolar or even nanomolar range; in some cases exhibiting greater potency than their natural counterparts.

## 7. Conclusions and Future Perspective

The exploration of novel anticancer agents, both natural and synthetic, is a rapidly advancing field. Chalcones, a class of organic compounds derived from either natural or synthetic sources, have attracted considerable attention for their anticancer properties. Numerous studies have demonstrated that chalcone derivatives and analogs exhibit potent anticancer activities across various PCa types, including CRPC. Extensive evidence underscores their capacity to influence various signaling pathways and cancer-related processes, enhancing their chemo-preventive potential. Furthermore, when used in combination with conventional chemotherapy, chalcones have shown promise in overcoming drug resistance and enhancing the therapeutic efficacy of existing treatments. Nevertheless, further in vivo studies are needed to provide a comprehensive understanding of the anticancer effects of chalcones in PCa. Additionally, pharmacokinetic studies are needed to determine the absorption, distribution, metabolism, and excretion (ADME) properties of these molecules and to further identify the best formulation and drug delivery systems for administering them. Furthermore, in vivo results must be validated through clinical trials to support the use of chalcones in patients with PCa. Since the safety of these molecules has not been extensively studied, determining their safety profile is of vital importance. Overall, the pharmacological effects of chalcones in PCa are noteworthy and offer promising potential to address the shortcomings of existing treatment strategies.

**Table 1:** Comprehensive list of chalcones with reported anti-prostate cancer (PCa) activity, including origin, experimental models, inhibitory concentrations (IC_50_), biological effects, and associated molecular targets/pathways.

No.	Chemical Structure	Origin	Research Model	Inhibitory Concentration (IC_50_)	Biological Effect	Target Pathway/Protein	Reference
**1**	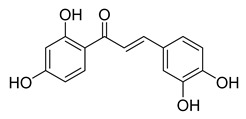	Natural	Cell-line(s): CWR22Rν1, LNCaP, PC3, and DU145 Animal model: xenograft mice model	N/A	Suppressed cell proliferation Promoted apoptosis Induced cell cycle arrest at the G2/M phase Suppressed cell motility, invasiveness, and angiogenic activity Reduced tumor growth in vivo	↓ VEGF, MMP-9, NF-κB, RANKL, IκBα kinase, *p*-IκBα, and PSA	[141,208,209]
**2**	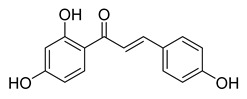	Natural	Cell-line(s): PC-3 DU145 LNCaP 22RV1	19.6 µM 23.3 µM 15.7 µM 36.6 µM	Suppressed growth and proliferation of cancer cells Induced cell cycle arrest at S and G2/M phases Promoted apoptosis Impaired mitochondrial membrane potential Inhibited EGF-stimulated cell invasion and migration Reduced tumor growth in vivo	↑ GADD153 Blocked ErbB3 signaling: ↓ *p*-ErbB3, ErbB3, p85, PSA, *p*-Akt ↓ VEGF, JNK ↑ TIMP-2	[98,126,143,150,170,210,211,212]
			Animal model: PC-3 xenograft mice model				
**3**	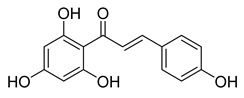	Natural	Cell-line(s): PC3 22RV1 PNT1A	For all cell lines ≥ 1 µM	Inhibited cell growth	N/A	[213]
**4**	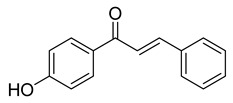	Synthetic	Cell-line(s): PC-3	6.19 μM	Inhibited cell proliferation	N/A	[214]
**5**	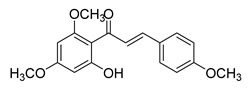	Natural	Cell-line(s): PC3, DU145 22Rv1 PrECs PrSCs Animal models: TRAMP mice model; FVB/N mice	22.86 μM N/A N/A >80 μM >80 μM	Selectively inhibited the growth of PCa cells with a pronounced effect on pRb-deficient cell lines Induced apoptosis and cell cycle arrest at G2/M phase Suppressed spheroid formation in CD44+/CD133+ CSCs. Suppressed tumor growth in TRAMP mice Dietary feeding of FKA inhibited tumor growth without causing any detrimental effects on the function of major organs in FVB/N mice.	↓ Tubulin polymerization ↑ Glutamine metabolism, ↓ intracellular glutamine, glutamic acid and proline ↓ GSS, ↑ GSTP1, ↓ GSH, ↑ ROS, ↑ apoptosis Proteasome-dependent and ubiquitination mediated Skp2 degradation: ↓ Skp2 ↑ p27/Kip1, ↓Nedd8-Cullin1, ↓ Nedd8-UBC12 ↓ Nanog, Oct4, CD44 ↓ Ubc12 neddylation, c-Myc, and keratin-8	[132,215,216,217]
**6**	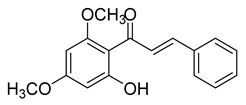	Natural	Cell-line(s): LNCaP DU145 PC-3 LAPC4	48.3 µM 3.9 µM 6.2 µM 32 µM	Suppressed growth of cancer cells Induced apoptosis Reduced tumor growth in vivo	↑ DR5, Bim, Puma ↓ XIAP, survivin ↓ Nedd8-Cullin1, Nedd8-UBC12 ↓ Skp2 (Increases Skp2 degradation in a ubiquitin and proteasome dependent manner) ↑ p21, p27	[101,218]
			Animal model: DU145 xenograft mice model				
**7**	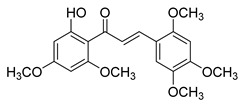	Natural	Cell-line(s): PC3 DU145 LNCaP RWPE-1 C4-2 PC3-PTX DU145-PTX	>50 µM in all cell lines	Combination therapy Rubone+ paclitaxel: Overcame paclitaxel chemoresistance with no impact on the proliferation of normal prostate cells. Inhibited PC3-TXR cell proliferation, spheroid formation in a 3D model, migration, and invasion. Inhibited the proliferation of CSCs Suppressed tumor growth in vivo	↑ miRNA-34a ↑ E-Cadherin, decrease SIRT1, decrease CyclinD1, and Bax ↑ Tap73, Elk-1	[186]
			Animal model: Nude mice				
**8**	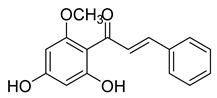	Natural	Cell-line(s): PC3 DU145 LNCaP	41.9 µM N/A N/A	Suppressed cancer cell proliferation Promoted apoptosis via DNA fragmentation Inhibited invasion and migration Increased cisplatin sensitivity Mitigated cisplatin-induced nephrotoxicity	↓ NF-κB ↓ *p*-JAK2, ↓ *p*-STAT3 (Tyr705) translocation & DNA binding ↓ VEGF, MMP-9, COX-2, XIAP	[180,219,220]
**9**	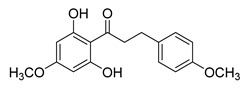	Natural	Cell-line(s): LNCaP	N/A	Enhanced TRAIL-induced cytotoxicity and apoptosis	N/A	[104]
**10**	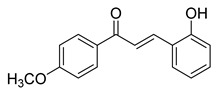	Synthetic	Cell-line(s): PC3	23.14 µM	Inhibited cancer cells proliferation	N/A	[221]
**11**	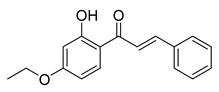	Synthetic	Cell-line(s): PC3	8.2 µM	Selectively inhibited cancer cell proliferation without impacting normal fibroblast cells Induced cell cycle arrest at the G2/M transition phase Triggered mitochondrial-dependent apoptosis	↑ Bax and ↓ Bcl-2 ↑ Caspase-3/7 activation	[99]
**12**	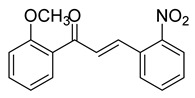	Synthetic	Cell-line(s): LNCaP	3.4 μM	Suppressed cancer cell growth	Stabilized the HSP90-AR complex in an androgen non-responsive state, blocking AR dependent transcription	[222]
**13**	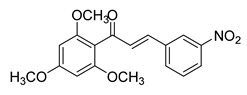	Synthetic	Cell-line(s): 22Rv1	N/A	Suppressed cancer cells proliferation Suppressed tumor growth in an ADT-resistant CRPC model without evident toxicities	Block Hsp40/Hsp70 chaperone axis → destabilize full-length AR → reduce the transcription of AR target genes	[223]
			Animal model: CRPC (22Rv1) xenograft mice model				
**14**	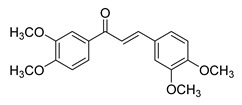	Synthetic	Cell-line(s): PC3 LNCaP	N/A	Suppressed metabolic activity Promoted macrophage polarization into an antitumor M1 phenotype	↑ mTORC1, IL-1β, TNF-α, and IL-10 of IL-4 stimulated macrophages ↓ NO level	[153]
**15**	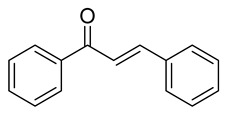	Synthetic	Cell-line(s): PC3 RWPE-1	1.1 µM 34.7 µM	Suppressed cancer cell growth Induced apoptosis through endoplasmic reticulum (ER) stress Elevated ROS production Inhibited tumor growth in nude mice	↑ IER1α sulfonation ↓ RIDD, mir-23b ↑ NOX4	[224]
			Animal model: Nude mice				
**16**	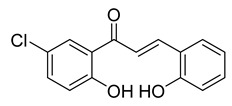	Synthetic	Cell-line(s): LNCaP PC3 DU145 Animal model: PC-3 xenograft mice model	3.74 µM 1.52 µM 4.5 µM	Suppressed proliferation of both AR-dependent and AR-independent PCa cells Induced SubG1accumulation Reduced tumor growth in vivo	Inhibited transcription of AR target genes Blocked DHT-induced growth Disrupted with the microtubule structure	[130]
**17**	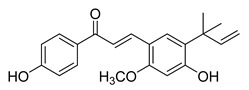	Natural	Cell-line(s): PC3 LNCaP	N/A	Promoted apoptosis via caspase pathway Arrested cell cycle at G2/M phase Induced autophagy	↓ p-Rb (S780) and E2F ↑ formation of acidic vesicular organelles ↓ mTOR	[123,152]
**18**	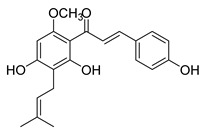	Natural	Cell-line(s): PC3 DU145 LNCaP C4-2 MCF-10a HLMEC	13.2 μM 12.3 μM N/A N/A	Selectively inhibited PCa cell proliferation compared to MCF-10A cells but not HLMEC endothelial cell Promoted apoptosis Enhanced sensitivity of TRAIL-resistant cancer cells	Induced mitochondrial depolarization ↑ cyt-c release ↓ NF-κB, Akt, mTOR, Bcl-2, and survivin	[100,102,188,195,225]
**19**	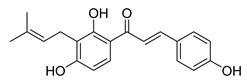	Natural	Cell-line(s): PC3 LNCaP	19.25 µM N/A	Suppressed cancer cells proliferation Promoted apoptosis via ROS Enhanced TRAIL-induced apoptosis	↓ TrxR1 activity → ↑ ROS and ER stress markers (GRP-78, ATF-4, XBP-1, CHOP, p-EIF2α) ↑ active caspase-3	[105,226]
**20**	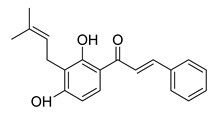	Natural	Cell-line(s): PC3	N/A	Induced G2/M cell cycle arrest	N/A	[227]
**21**	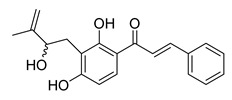	Natural	Cell-line(s): PC3 DU145	11 μM 7 μM	Suppressed cell proliferation Decreased colony formation Induced cell cycle arrest at G2/M phase	Disrupted tubulin structure	[133]
**22**	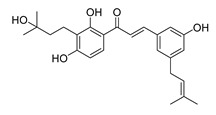	Natural	Cell-line(s): DU145	>10 µM	Suppressed cell proliferation	N/A	[228]
**23**	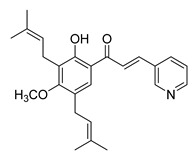	Synthetic	Cell-line(s): PC3 DU145 RWPE-1	4.67 µM 6.56 µM 5.00 µM	Inhibited the proliferation of cancer cells in a non-selective manner Induced apoptosis and GSDME-dependent pyroptosis Induced cell cycle arrest at sub-G1 Decreased metastasis, migration, and invasion Reduced colony formation Suppressed tumor growth in vivo	↑ active-Caspase-3, -8.-9, cyt-c, Bax/Bcl-2 ratio and PARP-cleavage) ↓ survivin, p-ERK1/2, p-P38/MAPK and MDM2 ↑ SHIP-1, and ↓ p110 and Gata-1 ↓ Cyclins D1 and D2, CDKs 2 and 6, and c-Myc, and ↑ P21cip1 & P27kip1 ↓ VEGF-1, ICAM-1, TGF-β2 and MMP-1 ↑ Fli-1 and Fli-1 target genes ↑ PKCδ, p-SAPK/JNK, and IL-6 GSDME cleavage → increases GSDME-N	[142,166,229]
			Animal model: PC3 xenograft mice mode				
**24**	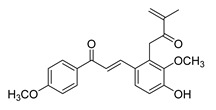	Natural	Cell-line(s): PC3	1.9 μM	Induced cytotoxicity	N/A	[230]
**25**	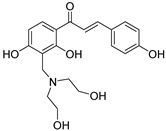	Synthetic	Cell-line(s): PC3	35.14 μM	Suppressed cancer cell proliferation	N/A	[231]
**26**	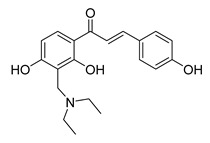	Synthetic	Cell-line(s): PC3	3.9 μM	Induced cytotoxicity	N/A	[232]
**27**	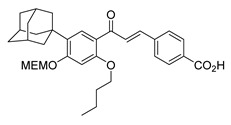	Synthetic	Cell-line(s): PC3	0.74 μM	Suppressed cancer cell growth/proliferation	Inhibited IkappaB kinase-beta (IKKβ)	[233]
**28**	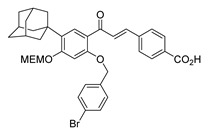	Synthetic	Cell-line(s): PC3	N/A	Inhibited cell viability Promoted apoptosis	↓ IκBα kinase α (IKKa) and IκBα kinase β (IKKβ)	[234]
**29**	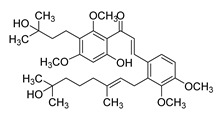	Synthetic	Cell-line(s): LNCaP PC3 DU-145	5.8 µM 9.2 µM 2.2 µM	Suppressed cancer cell growth and proliferation Induced G1 arrest	↓ p-RB, E2F-1, cyclin D1, cyclin E, CDKs 2 & 4, Cdc25A ↓ mTOR and survivin	[125]
**30**	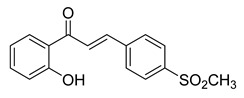	Synthetic	Cell-line(s): LNCaP PC3 DU145	15 μM 15 μM 20 μM	Reduced cell proliferation Induced apoptosis Sensitized PCa cells to TRAIL-induced apoptosis Promoted DNA fragmentation	↓ ∆ψm, Bcl-2NF-κB, COX-2 and p-Akt ↑ DNA fragmentation	[106,179]
**31**	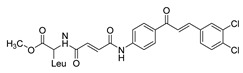	Synthetic	Cell-line(s): PC-3 LNCaP Animal model: mouse xenografts	29.5 μg/mL 21.4 μg/ml	Suppressed cell cycle progression, colony formation, invasion and migration Decreased neovascularization in chick embryos Significantly inhibited tumor growth in vivo	↓ MMP-p activity	[235]
**32**	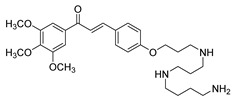	Synthetic	Cell-line(s): PC3 DU145	13.73 µM 8.86 µM	Suppressed cancer cell proliferation Induced apoptosis Arrested the cell cycle at G1 and G2 phases	↓ cyclin A2, Cdc2 and cyclin B1 ↑ p21, cleaved caspase-3, cleaved PARP and DNA fragmentation	[122]
**33**	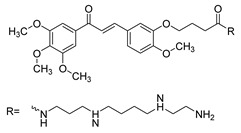	Synthetic	Cell-line(s): PC3 DU145	31.8 µM 28.5 µM	Inhibited cancer cell proliferation	N/A	[236]
**34**	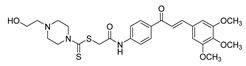	Synthetic	Cell-line(s): PC3	1.05 µM	Inhibited cancer cell proliferation Reduced colony formation Induced cell cycle arrest at G2/M phase Promoted DNA damage	↑ Caspase activation and ROS production ↓ ∆ψm and catalase activity Inhibited EMT (↑ E-cadherin and ↓ N-cadherin, Vimentin, activated-MMP2 and MMP9)	[124]
**35**	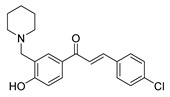	Synthetic	Cell-line(s): PC3	3.7 μM	Inhibited cell proliferation	N/A	[237]
**36**	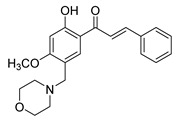	Synthetic	Cell-line(s): PC3	0.78 μg/mL	Inhibited cell proliferation	N/A	[238]
**37**	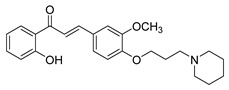	Synthetic	Cell-line(s): PC3 DU145	1.95 µM 2.73 µM	Suppressed cancer cell proliferation Promoted apoptosis	Disrupted tubulin polymerization and microtubule formation	[239]
**38**	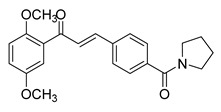	Synthetic	Cell-line(s): PC3	0.53 μM	Induced G1, S and G2/M phase arrests Promoted apoptosis	N/A	[240]
**39**	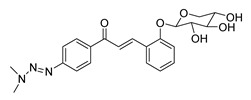	Synthetic	Cell-line(s): PC3	28.2 µM	Suppressed cancer cell proliferation	N/A	[241]
**40**	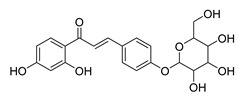	Natural	Cell-line(s): LNCaP PC3	19.35 µM N/A	Suppressed proliferation of AR-dependent PCa cells (LNCaP) without affecting AR-independent PCa cells (PC3) Induced G0/G1 cell cycle arrest Suppressed tumor growth in xenograft mice model	Disrupted AR signaling pathway ↓ cyclin D1 and CDK4	[119]
			Animal model: SCID mice				
**41**	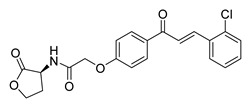	Synthetic	Cell-line(s): PC3 DU145 Normal GES-1	4.61 µM 3.24 µM 13.37 µM	Selectively suppressed proliferation of cancer cells Inhibited colony formation and cell migration Promoted apoptosis Sensitized PCa cells to TRAIL-induced apoptosis and growth inhibition (synergistic effect)	↑ DR5 ↑ Caspase3/7 activity	[107]
**42**	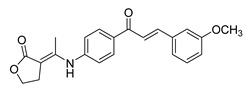	Synthetic	Cell-line(s): PC3 FL normal cells	69.92 µM 86.45 µM	Suppressed cancer cell proliferation	Formed covalent bonds with DNA	[189]
**43**	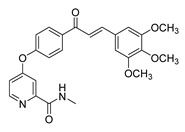	Synthetic	Cell-line(s): PC3	3.15 μM	Induced cytotoxicity	↓ VEGFR-2 and B-Raf Kinase activities	[242]
**44**	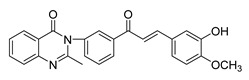	Synthetic	Cell-line(s): PC3 Animal model: Non PCa	54 μM	Suppressed cancer cell proliferation Promoted apoptosis Suppressed in vivo tumor growth without any detectable toxicity	N/A	[243]
**45**	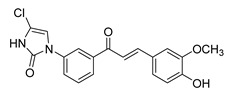	Synthetic	Cell-line(s): PC3	1.95 µM	Suppressed cancer cell proliferation	N/A	[244]
**46**	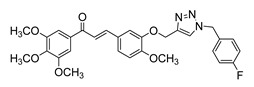	Synthetic	Cell-line(s): DU145	1.3 μM	Suppressed cancer cell proliferation Induced apoptosis and cell cycle arrest at G2/M phase	Blocked the polymerization of tubulin Downregulated ∆ψm Induced caspase-3 &9 activation	[131]
**47**	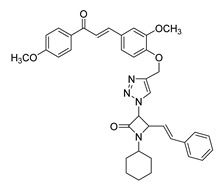	Synthetic	Cell-line(s): PC-3	67.1 μM	Suppressed cancer cell proliferation and growth	N/A	[245]
**48**	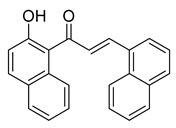	Synthetic	Cell-line(s): PC3 DU145	0.45 µM 0.64 µM	Suppressed cancer cell proliferation and growth Promoted apoptosis Arrested cells at prometaphase and metaphase	N/A	[118]
**49**	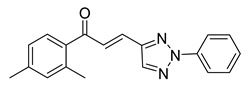	Synthetic	Cell-line(s): PC3	15.64 µM	Reduced cancer cells viability	N/A	[246]
**50**	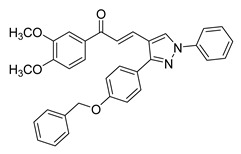	Synthetic	Cell-line(s): PC3	4.46 µM	Suppressed cancer cell proliferation Inhibited tubulin polymerization	N/A	[128]
**51**	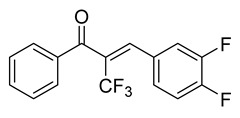	Synthetic	Cell-line(s): PC3 DU145 LNCaP Drug resistant cell lines Animal model: SCID mice	0.15 µM 0.19 µM N/A	Suppressed cancer cell proliferation Promoted apoptosis Arrested cell cycle at sub-G1 and G2/M phase Suppressed tumor growth in a xenograft mice model	↓ AR activity ↑ cleaved caspase-3, cleaved PARP ↓ Bcl-xl and Bcl-2	[120,121]
**52**	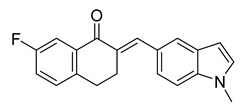	Synthetic	Cell-line(s): PC3	0.147	Suppressed cancer cell proliferation Promoted apoptosis Reduced invasion	Microtubule destabilization ↑ STMN1 Knockdown of STMN1 restored cancer cell viability	[247]
**53**	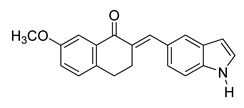	Synthetic	Cell-line(s): PC3 Normal lung bronchial epithelial cells	PC3 3 μM >10 µM	Suppressed cancer cell proliferation Induced apoptosis	N/A	[248]
**54**	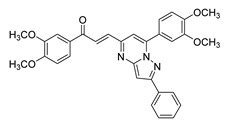	Synthetic	Cell-line(s): DU145	7.2 µM	Suppressed cancer cells proliferation	N/A	[249]
**55**	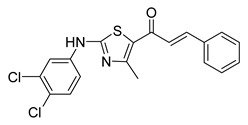	Synthetic	Cell-line(s): PC-3 Animal model: ICR mice bearing sarcoma 180	7.99 μM	Suppressed cell proliferation Moderate tumor inhibition in animal model Partially precipitated in the body, leading to obstruction in the mouse intestine	N/A	[250]
**56**	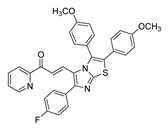	Synthetic	Cell-line(s): DU145	1.05 μM	Suppressed cancer cell proliferation Promoted apoptosis and accumulated cancer cells at G2/M phase	Inhibited tubulin polymerization via competing on colchicine binding site	[251]
**57**	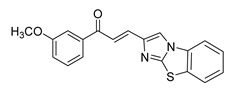	Synthetic	Cell-line(s): DU145	2.7 µM	Suppressed cancer cell proliferation	Blocked Microtubule assembly	[252]
**58**	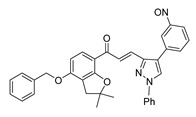	Synthetic	Cell-line model: PC3	5.9 μM	Suppressed cancer cell proliferation	N/A	[253]
**59**	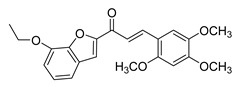	Synthetic	Cell-line(s): PC3	N/A	Suppressed cancer cell growth and reduced cell viability by approximately 85% at 20 μM Promoted apoptosis	↑ Caspase3/7 activation ↓ ∆ψm	[254]
**60**	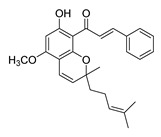	Natural	Cell-line(s): PC3	27.95 µM	Suppressed cancer cell proliferation	N/A	[255]
**61**	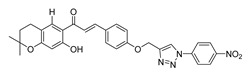	Synthetic	Cell-line(s): DU145	29.9 μM	Suppressed cancer cell growth	N/A	[256]
**62**	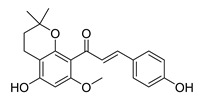	Natural	Cell-line(s): PC3	10.7 µM	Suppressed cancer cell proliferation	N/A	[257]
**63**	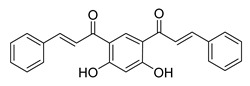	Synthetic	Cell-line(s): DU145	1.70 μM	Suppressed cell proliferation	N/A	[258]
**64**	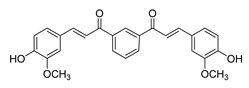	Synthetic	Cell-line(s): LNCaP PC3	5.04 μM 4.15 μM	Promoted cancer cell death and apoptosis	↓ proteasomal activity → ↑ ubiquitinated proteins ↑ Bax, Caspase-3, cleaved PARP	[103]
**65**	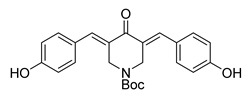	Synthetic	Cell-line(s): PC3 22RV1	22.9 µg/mL 17.1 µg/mL	Inhibited cell growth Promoted apoptosis	↓ NF-κB and KI67 ↑ Caspase3/7 activation	[190]
**66**	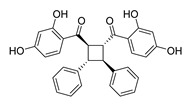	Natural	Cell-line(s): PC3	3.5 µM	Induced cytotoxicity	N/A	[259]
**67**	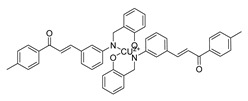	Synthetic	Cell-line(s): PC3	5.95 μM	Triggered cytotoxicity Exerted antioxidant activity	N/A	[260]
**68**	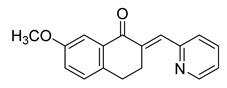	Synthetic	Cell-line(s): PC3 DU145	>10 µM <10 µM	Suppressed cancer cell proliferation	N/A	[261]
**69**	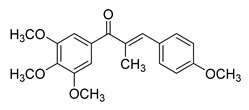	Synthetic	Cell-line(s): LNCaP PC3 DU-145 22Rv1 C4-2 Animal model: 22Rv1 xenograft model in male Nu/Nu nude mice	14–40 nM	Suppressed cancer cell growth Triggered apoptosis Induced cell cycle arrest Suppressed tumor growth of 22Rv1 xenograft models	↑ P53, p21Cip1 and cPARP ↓ Ki67 and ↑ TUNEL, P53 and P21	[127]
**70**	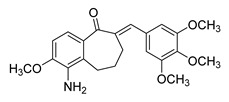	Synthetic	Cell-line(s): DU145 HUVEC	0.237 µM N/A	Selective inhibition of cancer cell proliferation with an SI >100 HUVEC cells.	N/A	[262]
**71**	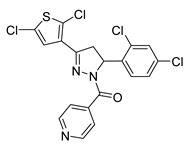	Synthetic	Cell-line(s): DU145	4.95 µM	Suppressed cancer cell proliferation	N/A	[263]

Abbreviations: PCa, prostate cancer; CRPC, castration-resistant prostate cancer; ADT, androgen-deprivation therapy; AR, androgen receptor; PSA, prostate-specific antigen; CSCs, cancer stem cells; IC_50_, half-maximal inhibitory concentration; EMT, epithelial–mesenchymal transition; VEGF, vascular endothelial growth factor; MMP, matrix metalloproteinase; NF-κB, nuclear factor kappa-B; TRAIL, TNF-related apoptosis-inducing ligand; DR5, death receptor 5; ROS, reactive oxygen species; CAM, chorioallantoic membrane; SI, selectivity index; N/A, not available or not assessed. Symbols: ↑ indicates upregulation/activation or increase; ↓ indicates downregulation/inhibition or decrease, → indicates a resulting effect, association, or progression from one event to another.

## Data Availability

No new data were created or analyzed in this study.
